# Molecular Evidence for the Thriving of *Campylobacter jejuni* ST-4526 in Japan

**DOI:** 10.1371/journal.pone.0048394

**Published:** 2012-11-07

**Authors:** Hiroshi Asakura, Holger Brüggemann, Samuel K. Sheppard, Tomoya Ekawa, Thomas F. Meyer, Shigeki Yamamoto, Shizunobu Igimi

**Affiliations:** 1 Division of Biomedical Food Research, National Institute of Health Sciences, Tokyo, Japan; 2 Department of Biomedicine, Aarhus University, Aarhus, Denmark; 3 Department of Zoology, University of Oxford, Oxford, United Kingdom; 4 Department of Molecular Biology, Max Planck Institute for Infection Biology, Berlin, Germany; Charité-University Medicine Berlin, Germany

## Abstract

*Campylobacter jejuni* is a leading cause of human gastroenteritis worldwide. This study aimed at a better understanding of the genetic diversity of this pathogen disseminated in Japan. We performed multilocus sequence typing (MLST) of *Campylobacter jejuni* isolated from different sources (100 human, 61 poultry, and 51 cattle isolates) in Japan between 2005 and 2006. This approach identified 62 sequence types (STs) and 19 clonal complexes (CCs), including 11 novel STs. These 62 STs were phylogenetically divided into 6 clusters, partially exhibiting host association. We identified a novel ST (ST-4526) that has never been reported in other countries; a phylogenetic analysis showed that ST-4526 and related STs showed distant lineage from the founder ST, ST-21 within CC-21. Comparative genome analysis was performed to investigate which properties could be responsible for the successful dissemination of ST-4526 in Japan. Results revealed that three representative ST-4526 isolates contained a putative island comprising the region from Cj0737 to Cj0744, which differed between the ST-4526 isolates and the reference strain NCTC11168 (ST-43/CC-21). Amino acid sequence alignment analyses showed that two of three ST-4526 isolates expressed 693aa- filamentous hemagglutination domain protein (FHA), while most of other *C. jejuni* strains whose genome were sequenced exhibited its truncation. Correspondingly, host cell binding of FHA-positive *C. jejuni* was greater than that of FHA-truncated strains, and exogenous administration of rFHA protein reduced cell adhesion of FHA-positive bacteria. Biochemical assays showed that this putative protein exhibited a dose-dependent binding affinity to heparan sulfate, indicating its adhesin activity. Moreover, ST-4526 showed increased antibiotic-resistance (nalidixic acid and fluoroquinolones) and a reduced ability for DNA uptake. Taken together, our data suggested that these combined features contributed to the clonal thriving of ST-4526 in Japan.

## Introduction

*Campylobacter jejuni* is one of the leading bacterial causes of foodborne gastroenteritis in humans and is responsible for an estimated 5–14% of the occurrence of human diarrhea worldwide, which translates into 400–500 million cases annually [1], [2]. This pathogen is typically located in the feces and gastrointestinal tracts of warm-blooded animals [3], [4], [5], [6], which are considered reservoirs for human infection. Human campylobacteriosis is most often sporadic; although poorly prepared food has often been implicated, it is generally difficult to determine the source since *C. jejuni* easily loses cultivability in food specimens under aerobic conditions. Therefore, a substantial effort has been made to isolate *C. jejuni* from a wide variety of reservoirs and type these strains using a number of methods, including amplified fragment length polymorphism (AFLP), *flaA* short variable region (SVR) sequencing, pulsed-field gel electrophoresis (PFGE), and multilocus sequence typing (MLST) [7]. MLST involves the phylotyping of bacterial isolates based on the DNA sequence comparison of a standardized panel of housekeeping genes [8]. As this method has advantages for accuracy and reproducibility of the data between different laboratories, MLST is one of the best approaches to understand the population genetics, host association, and seasonal or geographical variation of this pathogen [9], [10], [11], [12], [13], [14], [15], [16]. To date, 5,957 sequence types (STs) from 18,562 isolates have been obtained from MLST data, which have been deposited in the *Campylobacter* MLST database (http://www.plst.org/campylobacter) as for August 17, 2012. The clonal diversity and evolutional linkage of the STs could be visually analysed with eBURST [17], facilitating the evolutional analysis.

*C. jejuni* is a public health threat as one of the most common foodborne pathogens in Japan [18]. Results of a previous epidemiological study suggest that chicken and feedlot cattle are the main reservoirs for human campylobacteriosis [19]. Several molecular approaches, including PFGE, serotyping, and *flaA* genotyping, have provided a possible explanation for the widespread of this pathogen, which coincides with partial host association in Japan [20], [21], [22]. For example, serotypes O:2 and O:4 were common in human, poultry, and bovine isolates, whereas serotypes O:23, O:36, O:53 were common in human and bovine isolates in Japan [20]. The PFGE analysis also showed a partial similarity between poultry and human isolates [20]. In a more recent study, Yabe et al. [23] conducted a small-scale MLST analysis, revealing the presence of novel STs in *C. jejuni* isolated from poultry and humans in Japan. However, it remains unclear how and whether such host associations might be linked to human campylobacteriosis and whether there are geographic specifications that contribute to the thriving of this pathogen in certain populations within Japan.

**Table 1:** MLST scheme of *C. jejuni* isolates from different sources in Japan.

Source	No .isolate	Clonal Complex(CC)	%	STs*	%	Source	No.isolate	Clonal Complex(CC)	%	STs*	%
Human	100	CC-21	27.0	ST-50	9.0	Poultry	61	CC-21	16.4	**ST-4621**	6.6
				ST-8	3.0					ST-4526	4.9
				ST-21	4.0					ST-50	4.9
				ST-1360	1.0			CC-45	16.4	ST-3727	8.2
				ST-4526	7.0					ST-679	3.3
				ST-451	1.0					ST-45	3.3
				ST-4253	1.0					ST-845	1.6
				**ST-4614**	1.0			CC-48	11.5	ST-918	9.8
		CC-48	11.0	ST-48	7.0					ST-453	1.6
				ST-918	1.0			CC-443	8.2	ST-51	4.9
				ST-38	1.0					ST-440	1.6
				ST-453	1.0					**ST-4615**	1.6
				ST-918	1.0			CC-574	8.2	ST-305	8.2
		CC-42	6.0	ST-42	5.0			CC-353	6.6	**ST-4616**	3.3
				ST-447	1.0					ST-404	1.6
		CC-257	6.0	ST-257	4.0					ST-4052	1.6
				ST-361	2.0			CC-607	6.6	ST-4108	4.9
		CC-22	5.0	ST-22	4.0					ST-4600	1.6
				ST-1947	1.0			CC-354	4.9	ST-354	3.3
		CC-45	5.0	ST-45	4.0					**ST-4617**	1.6
				ST-3727	1.0			CC-460	3.3	ST-461	1.6
		CC-443	5.0	ST-51	2.0					ST-460	1.6
				ST-440	3.0			CC-22	3.3	ST-22	3.3
		CC-353	4.0	ST-4052	2.0			Others	14.8	**ST-4623**	6.6
				ST-353	1.0					ST-4389	3.3
				ST-2076	1.0					**ST-4618**	1.6
		CC-49	3.0	**ST-4624**	1.0					**ST-4620**	1.6
				**ST-4613**	2.0					**ST-4622**	1.6
		CC-354	3.0	**ST-4611**	1.0						
				**ST-4623**	1.0	**Cattle**	**51**	**CC-21**	**27.5**	**ST-21**	**7.8**
				ST-354	1.0					ST-806	15.7
		CC-45	2.0	ST-45	2.0					ST-50	3.9
		CC-460	2.0	ST-460	1.0			CC-42	19.6	ST-42	19.6
				ST-535	1.0			CC-22	9.8	ST-22	9.8
		CC-52	1.0	ST-52	1.0			CC-61	7.8	ST-61	7.8
		CC-61	1.0	ST-61	1.0			CC-48	7.8	ST-38	7.8
		CC-206	1.0	ST-46	1.0			CC-283	5.9	ST-4063	5.9
		CC-574	1.0	ST-305	1.0			CC-403	3.9	ST-933	3.9
		CC-658	1.0	ST-658	1.0			CC-257	2.0	ST-257	2.0
		Others	16.0	ST-922	6.0			Others	15.7	ST-58	15.7
				ST-2276	1.0						
				**ST-4612**	1.0						
				ST-407	4.0						
				ST-2328	2.0						
				ST-4390	2.0						

* Novel STs found in this study were shown in bold.

In the present study, we used an MLST approach to identify the population structure of *C. jejuni* isolated from human, poultry, and cattle across Japan for the first time. We identified a unique genotype, ST-4526, that thrived in poultry and human populations in Japan. A phylogeographical linkage analysis, in combination with comparative genome analyses, provided a possible explanation for this thriving.

## Materials and Methods

### Bacterial Isolates and Media

From 2005–2006, *C. jejuni* human clinical isolates (n = 100) were obtained from two prefectures, Tokyo and Osaka, in Japan. Poultry (n = 61) and cattle isolates (n = 51) were obtained from prefectures in Miyagi, Gunma, Akita, Kumamoto, Aichi, Hyogo, Yamaguchi (for poultry) or Oita and Hyogo (for cattle) during the same periods (Table S1, Fig. S1). The *C. jejuni* strain NCTC11168 [24] was used as a reference strain for comparative genomic and phenotypic studies. The *C. jejuni peb4* mutant [25] was used as a donor strain for the natural transformation efficiency assay. Bacterial isolates were grown on Mueller-Hinton (MH) agar or MH broth (Becton Dickinson, Franklin lakes, NJ, USA) at 37°C in a humidified CO_2_ AnaeroPack-Microaero gas system (Mitsubishi Gas Chemicals, Tokyo, Japan).

**Table 2:** Summary of the ST-4526 isolates determined in this study.

Isolate	Source	Isolation date	Isolation area	Serotype		PFGE group	LOS sialylation	Antibiotic resistance
				Lior	Penner			
H_0002	Human	May, 2005	Tokyo	LIO4	O:2	a	–	NA, CPFX, LVFX
H_0055	Human	Jun., 2005	Osaka	LIO27	O:2	d	–	NA, CPFX, LVFX
H_0059	Human	Aug., 2005	Osaka	LIO27	O:2	d	–	NA, CPFX, LVFX
H_0060	Human	Oct., 2005	Osaka	LIO4	O:2	a	–	NA, CPFX, LVFX
H_0089	Human	Jul., 2006	Osaka	LIO4	O:2	b	–	NA, CPFX, LVFX
H_0092	Human	Sep., 2006	Osaka	LIO4	O:2	b	–	NA, CPFX, LVFX
H_0097	Human	Oct., 2006	Osaka	LIO4	O:2	a	–	NA, CPFX, LVFX
P_0016	Poultry	April, 2005	Yamaguchi	LIO4	O:2	c	–	NA, CPFX, LVFX
P_0017	Poultry	June, 2005	Yamaguchi	LIO4	O:2	c	–	NA, CPFX, LVFX
P_0053	Poultry	July, 2005	Aichi	LIO4	O:2	c	–	NA, CPFX, LVFX

PFGE groups were corresponded to those in Fig. 3.

**Figure 1:**
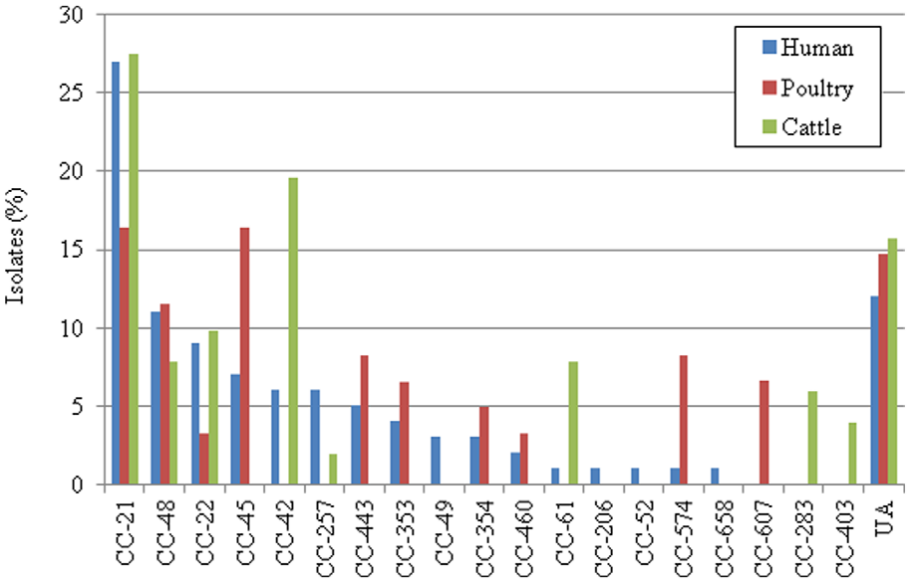
Frequency distribution of the *C. jejuni* clonal complexes (CCs) isolated from humans, poultry, and cattle in Japan (n = 212), which were classified into 62 sequence types (STs). UA represents isolates unassigned to any CCs.

### Multi-locus Sequence Typing (MLST) Analysis

Bacterial genomic DNA was extracted using a DNeasy Genomic Extraction kit (QIAGEN, Hilden, Germany) and stored at -20°C until further use. The PCR and cycle sequencing reactions were conducted according to the guidelines on the *Campylobacter* MLST database (http://pubmlst.org/campylobacter/). We confirmed the absence of the non-specific PCR amplicon using a 1% agarose gel. ExoSAP-IT was subsequently used to purify the PCR products according to the manufacturer’s instructions (Life Technologies, Carlsbad, CA, USA). For the sequencing reactions, we used both DNA strands in each allele with the BigDye terminator v. 3.1 Ready Reaction Cycle Sequencing kit on an ABI3730x DNA analyzer (Life technologies). The obtained sequences were assembled using CLC DNA Workbench software equipped with an MLST module (CLC bio, Aarhus, Denmark). The consensus sequences for each allele were assigned an allele number, a 7-locus (3,309 bp) sequence type (STs), and a clonal complex (CCs) through interrogation of the *Campylobacter* MLST database. Unassigned sequences were deposited in the database to obtain new allele or ST numbers according to the guidelines described on the website. The sequence data of each isolate were deposited in the database (each isolate ID is shown in Table S1).

**Figure 2:**
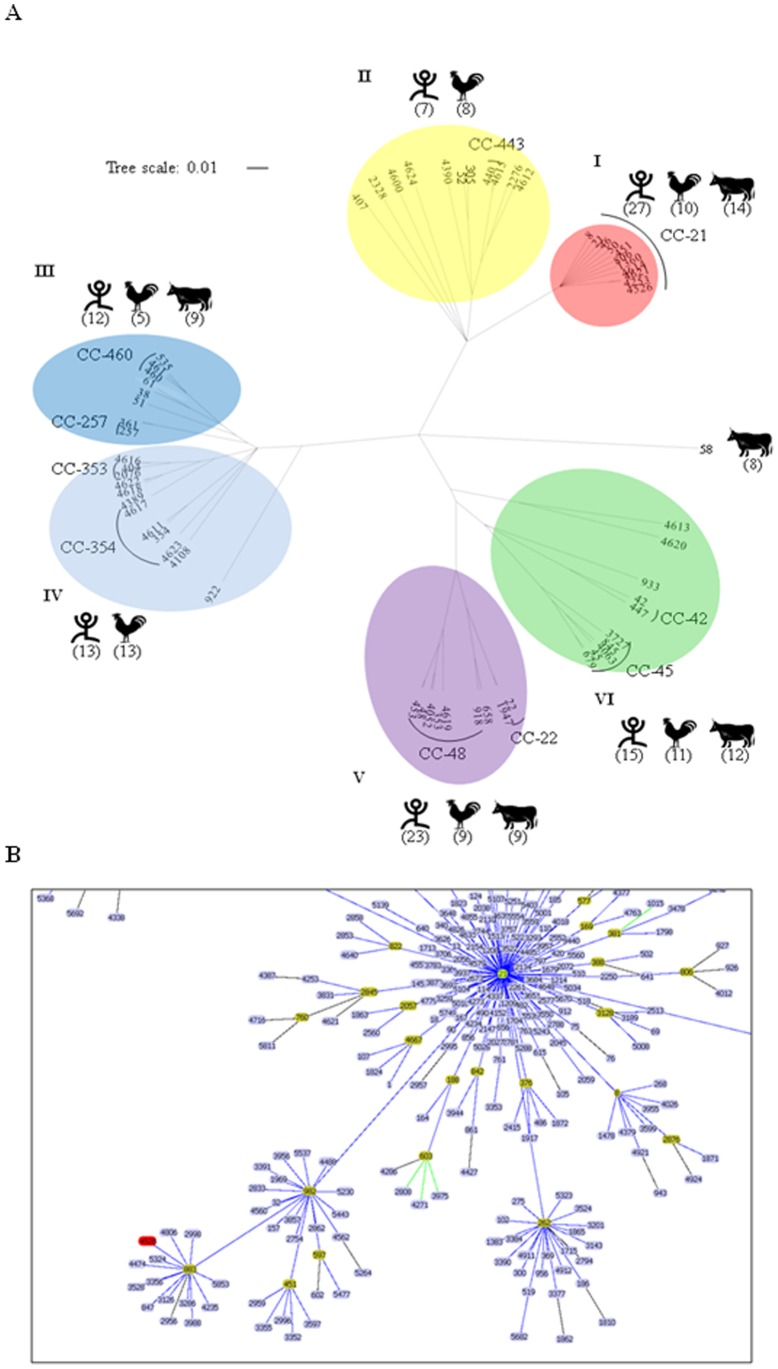
Genetic population and phylogeography of the *C. jejuni* MLST dataset. (A) The ClonalFrame analysis data for the 62 STs in the 212 *C. jejuni* isolates in Japan. The numbers of isolate sources per subgroup (cluster I to VI) are shown in the parenthesis, with illustrations (human, poultry, and cattle). (B) Enclosed graphic for the Global Optimal eBURST (goeBURST) analysis of *C. jejuni* CC-21 to assess the clonal lineage of ST-4526 (red) from the ancestor ST-21. The entire data is shown in supplemental Figure S1.

**Figure 3:**
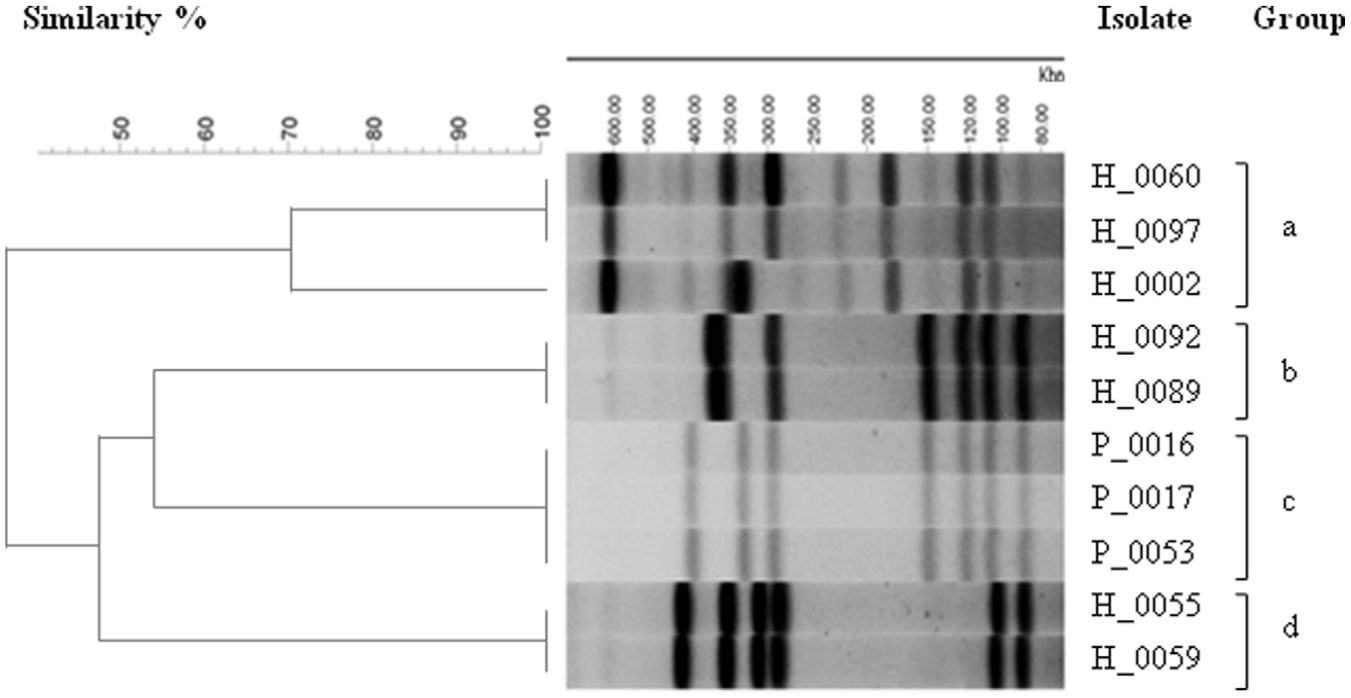
Pulsed-field gel electrophoresis (PFGE) analysis of *C*. *jejuni* ST-4526 isolates. The samples were *Sma*I-digested, followed by loading onto a 1% agarose gel. The isolates were divided into 4 groups, with a cut-off value of 70% similarity.

### Phylogenetic Analyses

The 7-locus allelic sequences were used to estimate the clonal genealogy of the STs using a model-based approach to determine the bacterial microevolution using ClonalFrame [26]. The output data were visualized using the iTOL (interactive Tree of Life) program (http://itol.embl.de/index.shtml) [27]. To assess clonal diversity and evolutional linkage, 7 allelic numbers for a total of 547 STs belonging to CC-21, were subjected to the Global Optimal eBURST (goeBURST, a java implementation of the eBURST algorithm proposed by Feil et al. [17]) [28].

**Figure 4:**
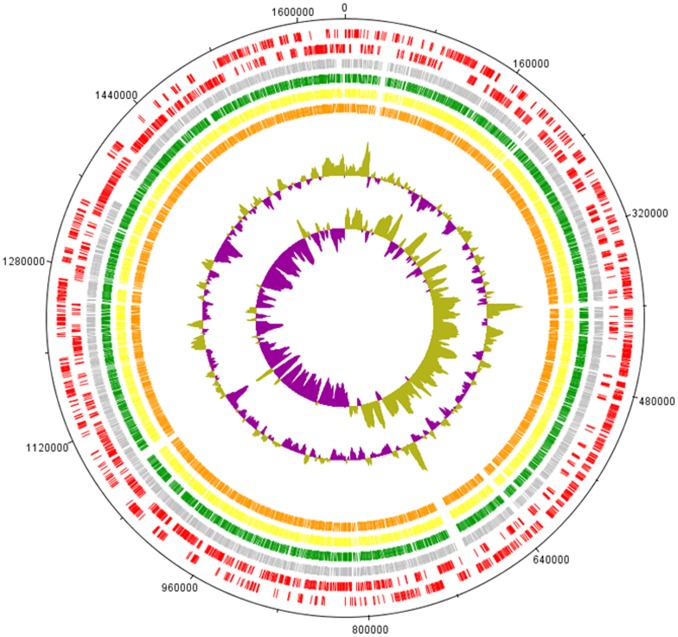
Comparative genome analysis of *C. jejuni* ST-4526. The CDSs of the reference genome of strain NCTC11168 (accession No. AL11168) are shown in the two outer rings (red, clockwise and counterclockwise). The subsequent rings depict RAST-annotated CDSs in the genomes of IA3902 (accession No.CP001876) (grey), H_0002 (green), H_0089 (yellow), and P_0053 (orange). The innermost ring depicts the GC content variation and GC skew from the mean (60%) of the reference genome.

### Pulsed-Field Gel Electrophoresis (PFGE)

All ST-4526 isolates (n = 10, Tables 1 and 2) were subjected to PFGE with *Sma*I endonuclease (New England BioLabs, Ipswich, MA, USA) using the CHEF Mapper system (Bio-Rad Laboratories, Hercules, CA, USA) as previously described [29]. The gel images were obtained using ethidium bromide stain. The electrophoretic patterns from PFGE were compared based on band position using FingerPrinting II software (Bio-Rad Laboratories) and derived using the Dice coefficient with a maximum position tolerance of 1%. The strains were clustered using the unweighted pair group (UPGMA) method with arithmetic averages according to the manufacturer’s instructions.

**Figure 5:**
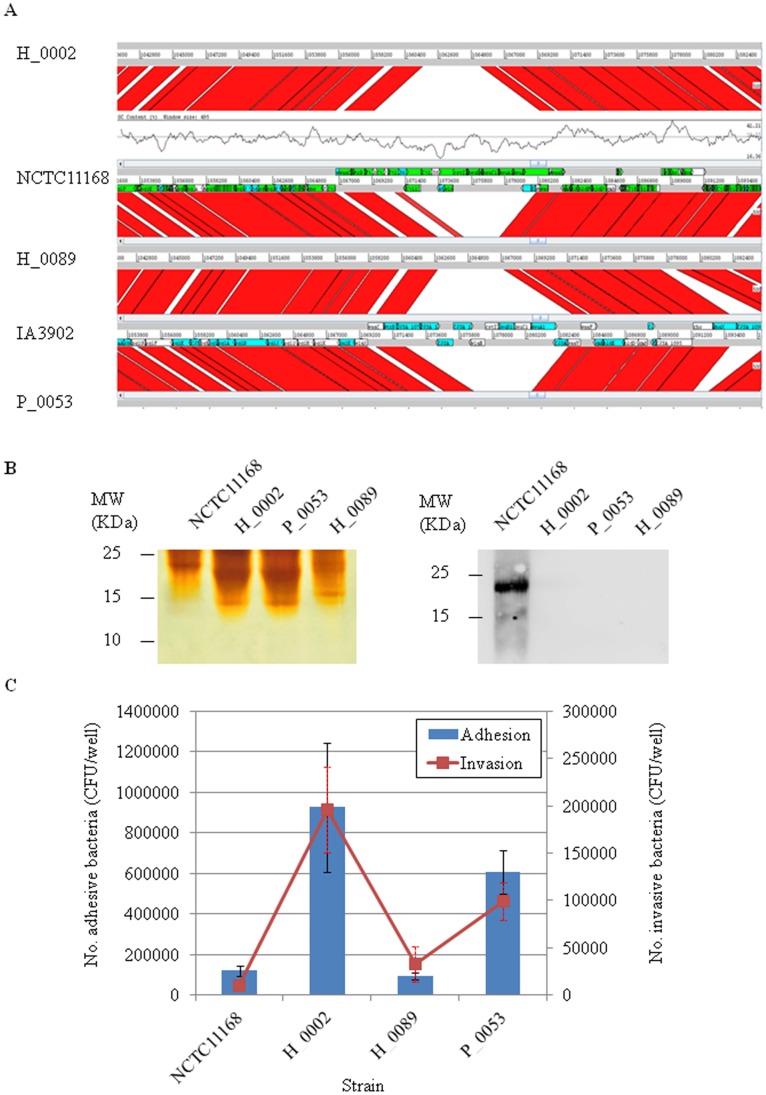
Genetic alteration in the lipooligosaccharide (LOS) modification locus in ST-4526. (A) Focused genetic alignment for the LOS locus among the NCTC11168, IA3902, H_0002, H_0089, and P_0053 strains, which was imaged using the Artemis Comparison Tool (ACT). (B) SDS-PAGE and detection of sialylation in *C. jejuni* LOS. The samples were extracted from NCTC11168, H_0002, H_0055, H_0089, and P_0053 strains and visualized using silver stain after loading onto 15% acrylamide gels (left panel). In parallel, the same samples on the acrylamide gels were subjected to western blot analysis using the GM1-reactive, HRP-conjugated cholera toxin B-subunit to detect sialylated LOS (right panel). (C) Cell adhesion and invasion properties of NCTC11168 and *C. jejuni* ST-4526 representatives. No adhesive or invasive bacteria were shown in either blue bar (means corresponding to the left Y-axis) or red lines (means corresponding to the right Y-axis), respectively.

### Genome Sequencing and Bioinformatics Analysis

Genomic DNA was extracted from three representative ST-4526 isolates (H_0002, H_0089, P_0053, which were selected randomly from the three different PFGE groups a, b, and c, respectively), using a Genomic tip-500 kit (QIAGEN, Hilden, Germany) and subjected to 8-kb paired-end pyrosequencing in a GS FLX+ system (Roche Diagnostics, Burgdorf, Switzerland) according to the manufacturer’s instructions. The sequence data (151,327, 173,181, and 167,955 reads for H_0002, H_0089, and P_0053, respectively) were assembled using the GS *De Novo* Assembler version 2.6 (454 Life Sciences, Branford, CT, USA). 77, 63, and 54 contigs were obtained (>500 bases) for the isolates H_0002, H_0089, and P_0053 at 41, 50, and 48-fold coverages, respectively. The draft genome sequences reported in this paper have been deposited in the GenBank database with accession number DRA000568.

The detection of open-reading frames and annotation of all draft genomes were automatically performed using the RAST service pipeline [30]. The Artemis Comparison Tool (ACT; http://www.sanger.ac.uk/resources/software/act/) was used for genome-wide comparisons of the nucleotide sequences [31]. Genome comparisons were also performed using a protein sequence-based bidirectional BLAST (Bi-BLAST) approach. The Island Viewer, which is a computational tool that integrates three different genomic island prediction methods, i.e., IslandPick, IslandPath-DIMOB, and SIGI-HMM [32], was used to identify the genomic islands. The results of all Bi-BLAST comparisons are shown in Table S6.

**Figure 6:**
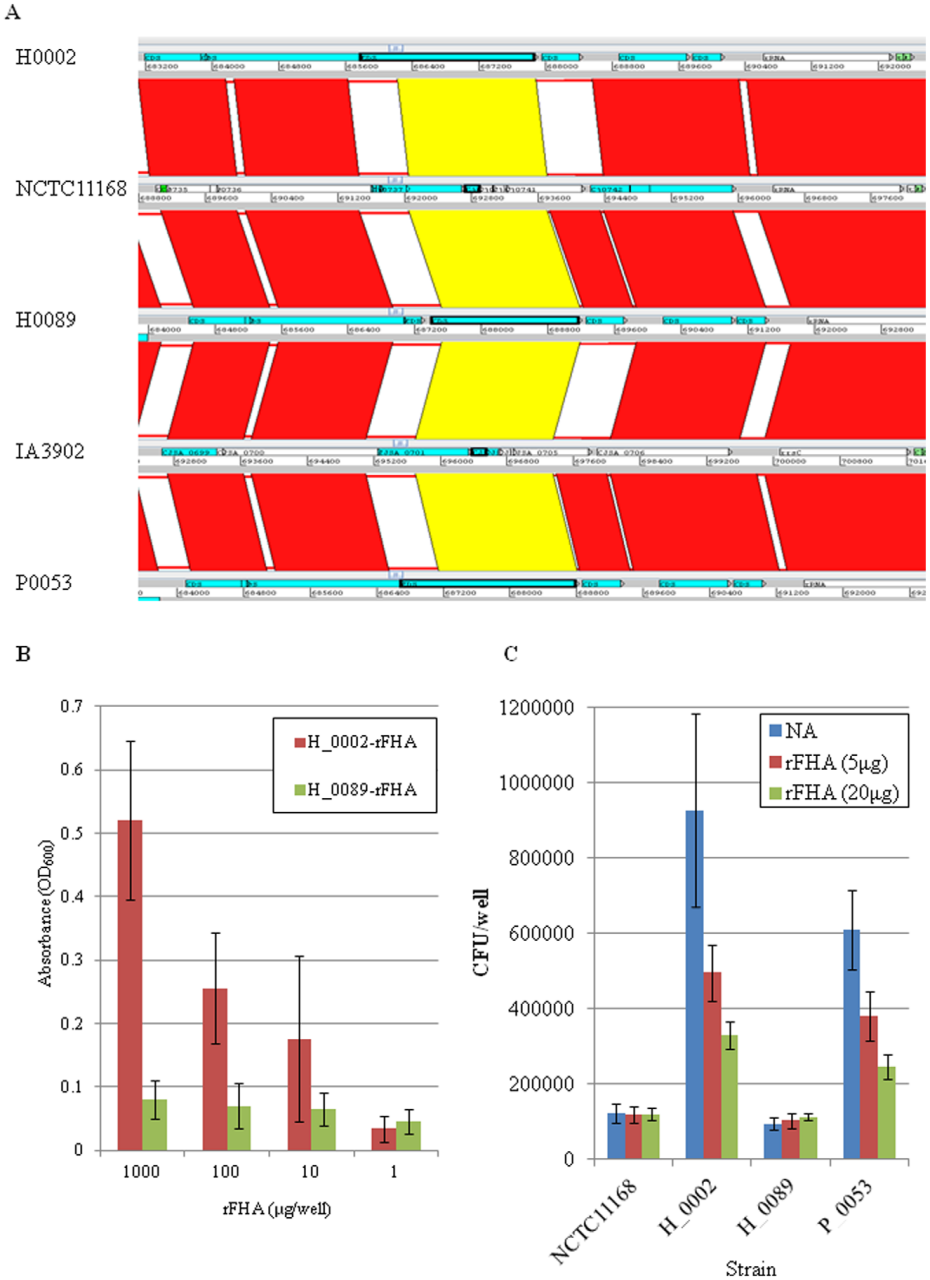
Sequence variation and host cell adhesive property of the FHA protein in *C. jejuni*. (A) Focused genetic alignment for putative island-like structures in the genomes of *C. jejuni* ST-4526, NCTC11168, and IA3902. The putative filamentous hemagglutination domain protein (FHA) is indicated with a bold-edge. (B) The FHA protein shows affinity to heparan sulfate *in vitro*. The tissue culture plates were pre-coated with heparan sulfate (5 µg/well). After washing with PBS, the plates were co-incubated with 100 µg and serial dilutions of recombinant FHA (rFHA) from isolates H_0002 or H_0089. The bound rFHA were solubilized with 0.1% SDS, and the protein concentrations were measured chronometrically using a Bradford assay. (C) The rFHA blocks the adhesion of FHA-positive *C. jejuni* on Caco-2 cells. The cells were pre-treated with 0, 5, or 20 µg of rFHA derived from H_0002 for 30 min prior to *C. jejuni* infection. The numbers of cell-associated bacteria were counted after gentle washing with PBS.

### LOS Isolation, SDS-PAGE, and Western Blot

Lipooligosaccharides (LOS) were prepared from *C. jejuni* grown on MH agar plates for 20 h at 37°C as described previously [33]. The samples were loaded onto 15% polyacrylamide gels and visualized using silver stain. The detection of LOS sialylation was performed using western blot analysis with cholera toxin B-subunit (Sigma Aldrich, St. Louis, MO, USA) as previously described [34].

**Table 3:** Genetic variations between the ST-4526 isolates and NCTC11168, determined by SNPs analyses.

Functional class	Gene (NCTC)*	Gene symbol	Description	No. variation*
Antibiotic resistance	Cj0003	*gyrB*	DNA gyrase B	5
	Cj1686c	*topA*	DNA topoisomerase I	5
	Cj1027c	*gyrA*	DNA gyrase A	1
DNA replication, recombination, repair	Cj0690c	–	putative restriction/modification (R/M) enzyme	39
	Cj1051c	*cjeI*	Restriction endonuclease S subunits (type I R/M)	21
	Cj0031	–	putative type II R/M enzyme	17
	Cj0139	–	putative endonuclease	9
	Cj1052c	*mutS*	recombination and DNA strand exchange inhibitor protein	6
	Cj0634	*dprA*	DNA processing protein A	5
	Cj1083c	–	putative nuclease	5
Flagellar-mediated motility	Cj1729c	*flgE2*	Flagellar basal body protein	22
	Cj1313	*pseH*	N-acetyltransferase specific for PseC product	20
	Cj1312	*pseG*	Nucleotidase specific for PseC product	18
	Cj1317	*pseI*	Pse synthetase	9

Representative SNPs in genes within the above functional categories were listed. For full SNP data, refer to Table S7.

* Gene orthologue in NCTC11168 was shown.

** No. unique variations found in the 3 ST-4526 isolates, with possible amino acid change, were shown.

### Cloning, Expression, and Purification of Recombinant FHA Protein in *E. coli*

The gene encoding the putative filamentous hemagglutination domain protein (FHA) was PCR-amplified from ST-4526 isolates H_0002 and H_0089 using primers (forward for H_0002: CAC.

CATGAAAAAGA TGAGTAAACATATAG, forward for H_0089: CACCATGGGAGTTTTGATT.

GGCAAAACAG, reverse: TAATTCTTCTTTAAAACTAGCGAAAT), directly cloned into the pBAD202 D-Topo vector, and transformed into *E. coli* LMG194 (Life Technologies). The expression of the His-tagged protein was induced in the presence of 0.1% L-arabinose according to the manufacturer’s instructions. The rFHA protein was purified using Ni-NTA resin (QIAGEN) according to the manufacturer’s instructions.

### Binding Affinity of rFHA to Heparan Sulfate

A total of 5 µg of heparan sulfate from porcine intestine (Sigma Aldrich) in sodium carbonate buffer (pH 9.6) was captured onto a 48-well tissue culture plate (TPP Techno Plastic Products AG, Trasadingen, Switzerland) at 4°C overnight. After washing with phosphate buffered saline (PBS, pH7.4) (Life technologies), 100 µg and serial dilutions of rFHA protein were added, and the plates were incubated at room temperature for 2 h. Following three washes with PBS, the bound proteins were solubilized in 20 mM Tris-HCl (pH 7.4) containing 0.1% SDS, and the protein concentrations were measured chronometrically using a Bradford assay.

### Cell Adhesion/Invasion Assay

Caco-2 cell adhesive/invasive assays were performed as described [35]. Briefly, the cells were infected with *C. jejuni* at an multiplicity of infection (MOI) of 100, and incubated for 6 h. The wells were gently washed three times with sterile PBS (pH 7.4) (Life technologies), and the cells were detached using 0.2% saponin in PBS for 20 min. The suspensions were subsequently plated onto MH agar to count the colony-forming units (CFU). To enumerate the numbers of intracellular bacteria, a gentamicin protection assay was conducted after the same incubation period as previously described [35]. To minimize the effect of motility on cell adhesion, the culture plate was spun down (500×*g*, 3 min) immediately after the bacterial inoculation to synchronize the bacteria onto the cell surface. To examine the blocking effect of rFHA, the cells were pre-treated for 30min with 0, 5, or 20 µg of rFHA derived from the H_0002 isolate prior to *C. jejuni* infection. The numbers of cell-associated bacteria were counted after gentle washing with PBS.

### Antibiotic Susceptibility Test

Disk diffusion tests were conducted to determine the susceptibilities of the *C. jejuni* ST-4526 isolates to nalidixic acid (NA), ciprofloxacine (CPFX), levofloxacin (LVFX), erythromycin (EM), ampicillin (ABPC), gentamycin (GM), and chrolamphenicol (CM) according to the supplier’s instructions (Becton Dickinson).

### DNA Uptake and Natural Transformation Efficiency Assays

The DNA uptake assay was performed essentially as described [36]. Briefly, 0.1 µg of plasmid pRY108 DNA [37] labeled with [α-^32^P]dCTP was co-incubated with approximately 4.5–5.8×10^7^ CFU of *C. jejuni* MH-broth suspension for 30 min. After washing and DNase I treatment, the intrabacterial uptake of radioactivity was measured using a scintillation counter. The transformation efficiency was also assayed in parallel according to the method of Wang and Taylor [38]. Briefly, equal amounts of *C. jejuni* MH-broth suspension as prepared above were incubated with 1 µg of chromosomal DNA from the *C. jejuni peb4* mutant (carrying Cm-resistant cassette) [25] on MH agar supplemented with 5% horse blood for 6 h. The bacterial cells were subsequently resuspended in 1 ml of MH broth, and 50 µl of the bacterial suspension were spread onto MH agar plates supplemented with or without Cm (20 µg/ml). The numbers of colonies grown on each plate were comparatively measured to determine the transformation efficiency.

### Statistics

The START ver.2 program [39] was used to determine the number of informative and parsimony informative sites, MLST alleles for each gene, and polymorphic sites according to the website guidelines (http://pubmlst.org/software/analysis/start2/). The genetic distances and rates of synonymous (*dS*) and non-synonymous substitutions (*dN*) were calculated using the MEGA ver.5 (Molecular Evolutionary Genetic Analysis) program [40]. The MEGA software was also used to draw amino acid alignment for the FHA protein. The data from all phenotypic assays represent the means ± standard deviations (SD) from at least three independent experiments.

## Results

### Summary of the *C. jejuni* MLST Dataset

A total of 212 *C. jejuni* isolates from human (n = 100) and animal sources (61 poultry and 51 cattle) collected in Japan from 2005–2006 were subjected to MLST analysis targeting seven genes (*aspA*, *glnA*, *gltA*, *glyA*, *pgm*, *tkt*, *uncA*). A total of 185 isolates (87.3%) were classified into 62 STs with 18 clonal complexes (CCs), and 11 STs (n = 27, 12.7%) were not assigned to any CCs (Tables 1 and S1); 42.5% of the strains belonged to three predominant CCs: CC-21 (n = 51, 24.1%), CC-48 (n = 22, 10.4%), and CC-45 (n = 17, 8.0%). The *pgm* gene yielded the greatest number of alleles, with 23 different alleles (Table S2), and *uncA* retained the greatest synonymous means (123.8) and lowest *dN*/*dS* value (0.0106). The total site variation within each gene ranged from 3.0% (*gltA*) to 14.9% (*uncA*) (Table S2). The host-by-host characteristics are summarized below.

- Human clinical isolates
- The human clinical isolates generated 43 STs (16 CCs plus 4 singletons), of which six STs were novel types (ST-4611 to 4614, 4623, and 4624) (Table 1, Fig. 1). Most of these novel types were represented by a single strain, except for ST-4613 (Table 1). A majority of the human isolates (81%) belonged to 10 CCs (CCs-21, −48, −22, −45, −42, −257, −443, −353, −49, and −354), of which 27% belonged to CC-21 (Fig. 1, Table 1). Within CC-21, the three most prevalent STs were ST-50 (n = 9, 33.3%), ST-22 (n = 8, 29.6%), and ST-4526 (n = 7, 25.9%) (Table 1).
- Poultry isolates
- The poultry isolates fell into 29 STs (10 CCs plus 6 singletons). CC-21 and CC-45 shared the highest (16.4%) coverage (Fig. 1, Table 1), in which ST-4526, one of the predominant STs among isolates from humans, was also represented by three poultry isolates (Table 1). A total of 9 novel STs were identified (ST-4615 to 4623), of which 4 isolates were associated with ST-4623 (Table 1). The most predominant STs were STs-918 (n = 6, 9.8%), −305 (n = 5, 8.2%), −4621 (n = 4, 6.6%), and −4623 (n = 4, 6.6%) (Table 1).
- Cattle isolates
- The 51 cattle isolates fell into 11 STs with 8 CCs (with one singleton, ST-58). Similar to the human and poultry populations, CC-21 shared the highest coverage (27.5%), and accordingly, CC-61 and CC-42 covered 19.6% and 7.8%, respectively (Fig. 1, Table 1). ST-42 and ST-806 shared either 19.6% or 15.7% coverage, respectively (Table 1). ST-58 was identified in 7 cattle isolates (Table 1), the source of which was commonly found in the Oita prefecture (Table S1).

### Population Structure of the MLST Dataset

To reveal the population structure of the *C. jejuni* isolates, a ClonalFrame analysis was conducted. This phylogenetic program grouped the 62 STs into 6 clusters (I to VI) (Fig. 2A); one of the predominant CCs, CC-21, was grouped into Cluster I. No cattle isolates were included in Clusters II and IV, which frequently included CC-460, CC-257, CC-353, and CC-354 from human and poultry populations. ST-58 was only identified in the cattle population (n = 8) and showed a distinct lineage from the other STs. Clusters III, V, and VI were generated in three (human, poultry, and cattle) populations. Thus, these data revealed a partial host association of distinct CCs/STs.

**Figure 7:**
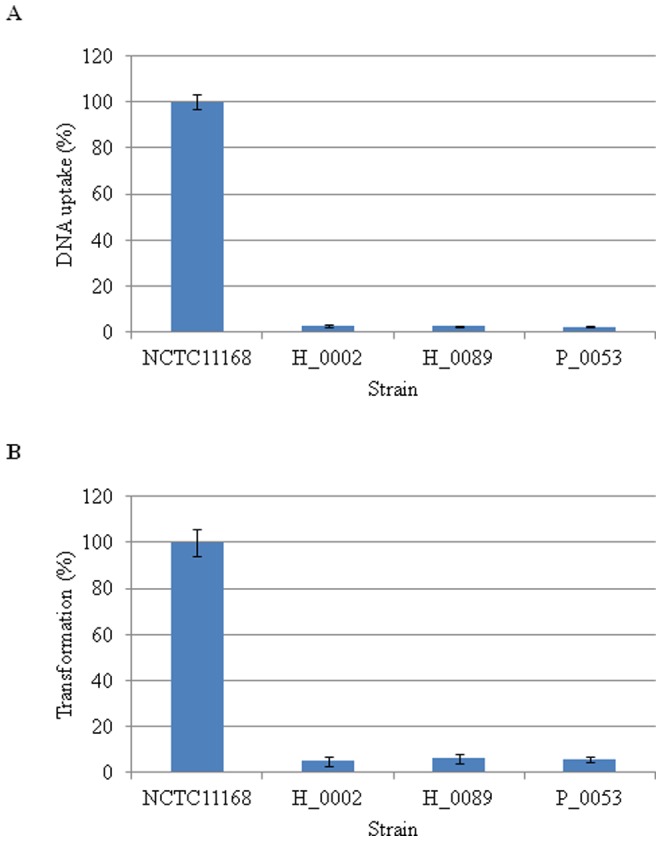
*C. jejuni* ST-4526 exhibits a reduced the ability for DNA uptake and natural transformation. (A) The bacterial cells were co-incubated with P^32^-labeled pRY108 DNA, and the intrabacterial radioactivity was measured using a scintillation counter. The means in the NCTC11168 strain were set to 100% for the comparative analysis. (B) The natural transformation efficiency was assayed. The appearance of the Cm-resistant clones was normalized to the total number of colonies on the plates without antibiotics, and the test strains were further normalized to the means in the NCTC11168 strain, which were set to 100%.

### Evolution Linkage of ST-4526 within CC-21

The MLST analysis revealed that ST-4526 was represented in 7 human and 3 poultry isolates, although their isolation areas and dates were diverse (Tables 1 and 2). Therefore, a search for this ST in the *Campylobacter* MLST database led to the identification of a single poultry isolate that was also isolated in Japan (Isolate ID 10249). For a more comprehensive analysis of the possible patterns of evolutionary descent, the goeBURST (global optimal eBURST) analysis [28] was carried out using a total of 547 STs belonging to CC-21 deposited on the *Campylobacter* pubMLST database (as of 2, August, 2012). This approach resulted that the ST-4526 showed one of the most distant lineage from the original (founding) ST-21 genotype, together with the genotypes originated only from human and poultry (ST-883 and the derivatives, Fig. 2B). Other 13 STs reported only from Japan (i.e. ST-4253, ST-4387, ST-5233) were classified into different clusters (Fig. S2). Together, these data support the idea that ST-4526 might be distinctly evolved from the ancestor.

### Summary of Genomic Features of *C. jejuni* ST-4526 Strains

Given the potent thriving and unique evolutional lineage of ST-4526, all ST-4526 isolates from human and poultry (n = 10) were macrogenotyped using pulsed-field gel electrophoresis (PFGE) analysis to determine their genomic similarities. As shown in Fig. 3, these isolates were divided into 4 groups (a–d), in which 3 poultry isolates (P_0016, P_0017, P_0053; group c) were closely related to the human isolates H_0089 and H_0092 that belonged to group b. To further clarify the genomic similarities and characteristics that might account for the thriving of these bacteria, three representative isolates (H_0002, group a; H_0089, group b; P_0053, group c) were genome sequenced, resulting in contigs comprising 1,625,252 bp (H_0002), 1,627,144 bp (H_0089), and 1,626,331 bp (P_0053). The sequences were subsequently processed with the RAST annotation pipeline [30], which annotated 1,836 to 1,865 features (Tables S3–S5). The three ST-4526 genomes were compared with the genomes of NCTC11168 (ST-43/CC-21, accession No. AL11168) and IA3902 (ST-8/CC-21, accession No. CP001876). DNA Plotter depicted the comparative genome circular maps for the genomes discussed above (Fig. 4).

### ST-4526 Expresses Asialylated forms of Lipooligosaccharides (LOS)

The genome alignments using ACT (Artemis Comparison Tool) combined with a Bi-BLAST comparison of *C. jejuni* ST-4526 strains with strains NCTC11168/IA3902 clearly showed significant overall synteny between the five genomes. However, the LOS modification locus was not conserved (Cj1137c to Cj1144c) (Fig. 5A, Table S6). In contrast to strain NCTC11168, the LOS of the 3 ST-4526 isolates did not react with the cholera toxin, a ligand for GM1-oligosaccharide structures (Fig. 5B). Sialylated LOS structure is known to contribute to cell invasion [41] and gastrointestinal colonization in poultry [42]. The Caco-2 cell adhesion/invasion assays indicated, however, that the H_0002 and P_0053 isolates exhibited greater cell adhesion and invasion properties than the H_0089 and NCTC11168 isolates (Fig. 5C), providing an idea that the LOS differences did not essentially contribute to cell adhesion/invasion, hence certain determinant(s) might preferentially promote cell adhesion and invasion in H_0002 and P_0053.

### ST-4526 Expresses a Putative Adhesin Protein with a Filamentous Hemagglutination Domain (FHA)

The Bi-BLAST analyses further revealed that the region from Cj0738 to Cj0752 was highly diverse between ST-4526 and NCTC11168 (Table S6). The Island Viewer [32] subsequently predicted that the three ST-4526 isolates contained a putative island comprising the region from Cj0737 to Cj0744, which harbored genes which differed between ST-4526 and NCTC11168/IA3902 (Fig. 6A). The amino acid alignment analyses showed that the Cj0742-homologue filamentous hemagglutination domain protein (FHA) was expressed in isolates H_0002, P_0053 and strain CF93-3 (accession No. CJJCF936_0827), which encoded a 693 aa protein with 100% sequence similarity; notably, this protein was truncated in H_0089 and NCTC11168/IA3902, with 585 aa (lacking N-terminus) and 358 aa (lacking C-terminus), respectively (Fig. S3). Other strains also exhibited distinct aa sequences, ranging from 358aa (strains 84-25 and DFVF1099) to 633aa (strain 1213) (Fig. S3).

FHA functions as an adhesin in *Bordetella pertussis*, showing binding affinity to carbohydrates, such as heparan sulfate, distributed on epithelial cell surfaces [43]; therefore, we produced recombinant FHA (rFHA) protein in *E. coli* (Fig. S4) and measured its affinity to heparan sulfate. The results showed that the rFHA from the H_0002 isolate showed greater binding affinity to heparan than the rFHA from the H_0089 isolate (Fig. 6B), indicating that the N-terminus region of FHA protein was required for this binding affinity. To further support the idea that FHA mediated bacterial adhesion on Caco-2 cells (Fig. 5C), the cells were pre-treated with rFHA prior to infection with H_0002 and P_0053 isolates, and a significant reduction in their cell adhesion levels was observed (Fig. 6C). Thus, these data indicated that the ST-4526 isolates H_0002 and P_0053 expressed functional FHA, which functions as an adhesin.

### ST-4526 Increases Antibiotic Resistance and Reduces DNA Uptake/Recombination

The single nucleotide polymorphism (SNP) analyses with NCTC11168 additionally revealed unique genetic variations in the genes for antibiotic resistance, DNA uptake/recombination, and flagellar motility in the ST-4526 (Table 3, Table S7).

#### (i) Antibiotic resistance

The ST-4526 isolates exhibited SNPs within DNA topoisomerase genes, including *gyrA* at Thr86Ile; *gyrB* at 5 positions, including Thr225Ile, Asp309Glu, Ile318Val, Ser667Asn, and Ser677Gly; and *topA* at 5 positions, including Ala360Ser, Val401Ala, Thr489Ile, Ser607Gly, and Ala212Val (Table S7). The single base mutation in *gyrA* at Thr86 plays a role in increasing the resistance of nalidixic acid (NA) and fluoroquinolones in *Campylobacter* [44], [45]. Accordingly, a disk diffusion test confirmed the resistance of the ST-4526 isolates to NA and fluoroquinolones (CPFX and LVFX) (Table 2).

#### (ii) DNA uptake and transformation efficiency

*C. jejuni* takes up exogenous DNA via the type II secretion system (T2SS), which is likely to affect genomic plasticity in this pathogen [36]. The Bi-BLAST analyses revealed a reduced amino acid similarity in the Cj1474c gene product (CtsD, a putative type II secretion system D protein) compared with that in NCTC11168 (% similarity at 65.5–66.3%) (Table S6). In addition, many variations were observed in the restriction-modification (R-M) system genes (Cj0690c, 39 SNPs, Cj0031, and 17 SNPs) (Table 3, Table S7), which are also involved in a barrier against foreign DNA and bacteriophages [46], [47]. We thus reasoned that ST-4526 might lose the ability for uptake of exogenous nucleotides, thereby reducing recombination. The ST-4526 isolates showed a more reduced (2.0–3.0%) uptake of exogenous ^32^P-labeled plasmid DNA than the NCTC11168 strain (Fig. 7A). Similarly, ST-4526 also exhibited reduced transformation efficiency compared with NCTC11168 (5.0–6.1% relative efficiency, Fig. 7B). Taken together, our data indicated that ST-4526 exhibited a loss-of-function for the DNA uptake and recombination.

#### (iii) Flagellar-mediated motility

A total of 4 genes for flagellar-mediated motility, which is prerequisite for establishing host colonization during an early course of infection [48], [49], [50], also showed sequence variations between ST-4526 and NCTC11168, with 22 SNPs in Cj1729c (*flgE2*), 20 SNPs in Cj1313 (*pseH*), 18 SNPs in Cj1312 (*pseG*), and 9 SNPs in Cj1317 (*pseI*) (Table 3, Table S7). The soft agar motility assay, however, showed that the ST-4526 isolates were motile, with no significant difference with NCTC11168 (Fig. S5). Thus, we showed that the nucleotide variations did not affect the motility phenotype in ST-4526.

## Discussion

Genetic attribution of bacterial genotypes has become a major tool in the investigation of the epidemiology of campylobacteriosis and has implicated retail chicken meat as the major source of human infection in several countries [51], [52]. In this study, we analyzed the population genetics of *C. jejuni* from different sources and areas in Japan using a MLST approach. We also attempted to understand the molecular basis underlying the unique and widespread predominance of ST-4526 in Japan using comparative genomic analyses. We identified several genomic traits beneficial for host adaptation, which seemingly accounted for the thriving of this genotype.

Many campylobacters are commensals that pass through a wide range of warm-blooded hosts and insects. Within farm animals, certain MLST lineages were identified in chickens and cattle, with different frequencies, whereas several CCs (i.e. CC-21) were identified at high frequencies in both sources [53]. CC-21 and -45 were the most frequent CCs in humans [54], [55], whereas CC-21, CC-45, and CC-574 were predominant in the poultry population in Great Britain from 2003 to 2006 [56]. In addition, Kwan et al. [57] reported the predominance of CC-61, CC-21, and CC-42 among cattle isolates in the United Kingdom. Our data are consistent with the finding for host assignment discussed above, regardless of geographic distance. In a recent large-scale MLST data analysis, Sheppard et al. [58] demonstrated that host association is more robust than geographic associations.

The results of the ClonalFrame analysis also revealed the presence of the rare genotype ST-58. In this study, however, this genotype was detected in 8 cattle isolates derived from the same location (Oita prefecture), although they were collected at different periods. Therefore, we could not rule out that this genotype might be adapted to the cattle population at a particular geographical location (farms). The additional collection of cattle isolates from different locations, including this area, would help to ascertain whether ST-58 also is a cattle-adaptive genotype.

There is an increasing evidence for the local and international transmission of host-associated lineages between food animal species [58]. In particular, CC-21, a widely distributed group, showed high genomic similarity regardless of origin [59]. We observed the frequent distribution of ST-4526 within CC-21 between the human and poultry populations of our collection. This genotype has not been previously reported from other countries; therefore, we examined the evolutionary linkage of this genotype within CC-21, which revealed a distinct lineage of this genotype from frequent genotypes worldwide. It remains unknown whether such phylogenetic distances represent bacterial traits for adaptation or host colonization. Lang et al. [60] showed that host species was a significant factor in explaining the genetic variation and macrogeography between Western Europe and the USA, and geographic variation of *C. jejuni* genotypes have been previously reported in Great Britain [56] and the USA [61]. The evolutionary linkage data herein thus suggested that ST-4526 genotype might be evolved in the Asian geography for adaptation. Comparative genomics between the closely related STs in goeBURST analysis (i.e. ST-4526 vs ST-883) would clarify whether the housekeeping genes-based clonal analysis might represent bacterial fitness for environmental survival or host infection, host specificity.

The availability of genome sequences facilitates a qualitative advance in our understanding of the epidemiology, ecology, and molecular biology of various organisms. To characterize the genomic features that account for the thriving of ST-4526, a comparative genomic analysis was performed for three ST-4526 isolates (H_0002, H_0089, and P_0053). This genotype showed (asialylated) LOS forms distinct from those of NCTC11168, although the latter showed forms similar to the widespread genotypes within CC-21. Habib et al. (2008) [62] showed a strong (85%) correlation between CC-21 and the sialylated class C LOS, indicating a potential role for class C LOS in the evolution of CC-21 that might be of particular importance in the poultry meat-related transmission of *C. jejuni* to humans.

We also observed variations in island-like structures, which included putative adhesin, in the genome of *C. jejuni*; i.e., fig|6666666.15583.peg.771 to fig|6666666.15583.peg.779 in H_0002 (Table S3), which showed less similarity to NCTC11168 (corresponded to the locus Cj0736-Cj743) (Table S6). The amino acid alignment revealed the truncation of the putative filamentous hemagglutination domain protein (FHA) among the *C. jejuni* strains. The recombinant FHA protein showed dose-dependent binding affinity to heparan sulfate, which competitively inhibited bacterial adhesion, suggesting a putative role for this gene product as an adhesin. Future studies will be focused on the in-depth biochemical characterization of this putative adhesin protein (i.e., determination of the active domain for cell adhesion, with screening of the host cell surface carbohydrates as interactive partners), which will be helpful for clarifying the biological role of this putative molecule during infection.

The SNP analyses in combination with a Bi-BLAST search revealed several genetic variations unique to the ST-4526 isolates; the polymorphisms in the topoisomerase genes (especially *gyrA*), could explain the increased resistance to NA and CPFX/LVFX, which is likely to confer an advantage for the spread and stability of this pathogen in poultry hosts as previously reported [63], [64]. This SNP was also identified in the genes for DNA uptake and recombination, and phenotypic or biochemical assays confirmed these reduced abilities. The genetic exchange of this pathogen was previously demonstrated in the chicken intestinal environment [65]. Nevertheless, the observed phenotypes in ST-4526 could thus explain the inability of this genotype to cause frequent genomic alterations, i.e., expanding the ability for clonal spread in human and poultry populations in Japan. It remains unknown how ST-4526 acquired such phenotypes; one possible explanation is that ST-4526 might evolve during the colonization of the ancestor in the poultry host, as passage in the chicken gut could alter the genetic and phenotypic traits of this pathogen [66]. With consideration for unique phylogeny, future studies will attempt to clarify the potential role of farm animals in the genomic evolution in this pathogen.

The ST-4526 isolates were commonly serotyped to O:2. A previous study showed that serotypes O:2, O:4, O:37, and O:8 shared more than 45.7% of a total of 601 poultry isolates in Japan collected from 2001 and 2006 [22]. The predominance of these genotypes in both the human and poultry populations reported herein reinforced the need for continued research on the potential importance of poultry as reservoirs for these human pathogens. We are currently collecting human isolates at different periods to determine whether the thriving of the ST-4526 is temporal or continuous with geographical significance.

In summary, this study is the first to show the population structure of *C. jejuni* from different sources in Japan, which deciphered the potent thriving of the ST-4526 among human and poultry populations. The results of genomic and phenotypic analyses provided possible reasons for the wide spread of this genotype. Our continuous examination of the molecular basis underlying the genotypic alterations and phylogeography of this pathogen will provide further insight into the link between the evolution and ecological features of this pathogen.

## Supporting Information

Figure S1 **Japan geography for the source of** ***C. jejuni*** **isolates used in this study.**
(TIF)

Figure S2 **The goeBURST analysis of** ***C. jejuni*** **CC-21. A total of 547 STs belonging to the CC-21 were used to analyze possible patterns of their evolutionary descent.**
(TIF)

Figure S3 **Multiple alignment of putative filamentous hemagglutination domain protein (FHA) in** ***C. jejuni*** **ST-4526 representative isolates (H_0002, H_0089, P_0053), and those from 12 other** ***C. jejuni*** **strains (CF93-6, 1213, LMG23263, 1997-14, 1854, S3, 84-25, NCTC11168, IA3902, DFVF1099, M1, and 81116).** The conserved domain Architecture Retrieval Tool (CDART) (http://www.ncbi.nlm.nih.gov/Structure/exington/lexington.cgi) predicts the putative hemagglutination activity domain at 22 to 135 aa from the N-terminus of the H_0002 isolate (shown in bold).
(TIF)

Figure S4 **Cloning and expression of the recombinant filamentous hemagglutination domain protein (rFHA) from** ***C. jejuni*** **H_0002 and H_0089.** The putative FHA-encoding gene was PCR-amplified (section A), and ligated into pBAD202 D-Topo vector. Expression was induced under the control of L-arabinose in *E. coli* LMG194 strain. Both the purified rFHA proteins were loaded onto 10% acrylamide gel, visualized by CBB stain. M, molecular markers (section A, λ/*Hin*dIII; section B, Bio-Rad Precision plus Protein standard Dual color).
(TIF)

Figure S5 **Motility of** ***C. jejuni*** **ST-4526 isolates on soft agar plates. 5 µl aliquots of** ***C. jejuni*** **ST-4526 isolates (H_0002, H_0089, P_0053) and NCTC11168 cultures were spotted on 0.4% soft agar plates.** At 24 h post incubation, the diameters of halo around the spot were measured. The data showed means ± standard deviation (SD) from three independent testing.
(TIF)

Table S1 ***Campylobacter jejuni*** **isolates used in this study.** ^*^Novel STs found in this study were shown in bold. ^**^UA, unassigned to any CCs. ^***^Isolate ID, deposited onto the *Campylobacter* MLST database (http://pubmlst.org/perl/bigsdb/bigsdb.pl?db=pubmlst_campylobacter_isolates&page=query).
(XLS)

Table S2 **Summary of the MLST alleles statistics for the** ***C. jejuni*** **dataset in Japan.** Seven alleles obtained from a total of 212 *C. jejuni* isolates ( = 62STs) were statistically analyzed by START2 program, providing the allele frequency, and polymorphisms. ^*^ *dN*/*dS*: Mean non-synomous substitutions per non-sinoumous site (*dN*)/mean synonymous substitutions per suynomous site (*dS*). ^**^Sites are categorized by the degeneracy of the codon they belong to, rather than that of the individual site.
(XLSX)

Table S3 **Genomic annotation of** ***C. jejuni*** **H_0002 (Table S3), P_0053 (Table S4), and H_0089 (Table S5).**
(XLS)

Table S4 **Genomic annotation of** ***C. jejuni*** **H_0002 (Table S3), P_0053 (Table S4), and H_0089 (Table S5).**
(XLSX)

Table S5 **Genomic annotation of** ***C. jejuni*** **H_0002 (Table S3), P_0053 (Table S4), and H_0089 (Table S5).**
(XLSX)

Table S6 **Bidirectional BLAST (Bi-BLAST) comparisons for** ***C. jejuni*** **H_0002, H_0089, P_0053, IA3902 toward NCTC11168 strain.**
(XLSX)

Table S7 **Single nucleotide polymorphism (SNP) analysis for the ST-4526 isolates to the NCTC11168 strain.** Contig sequences of three ST-4526 isolates (H_0002, H_0089, P_0053) were used to search SNPs unique to ST-4526 by comparison with the genome of NCTC11168 strain using CLC Genomic workbench software.
(XLSX)
