# Supplementary figures and images for: Molecular Evidence for the Thriving of Campylobacter jejuni ST-4526 in Japan
Source: PLoS One. 2012 Nov 7;7(11):e48394. doi: 10.1371/journal.pone.0048394 (PMC3492356; doi:10.1371/journal.pone.0048394)

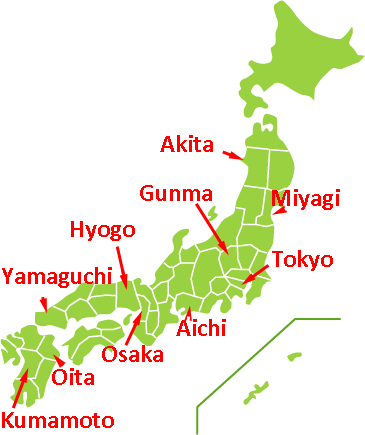

Supplement: Figure S1 — Japan geography for the source of C. jejuni isolates used in this study. (TIF) [file pone.0048394.s001.tif]

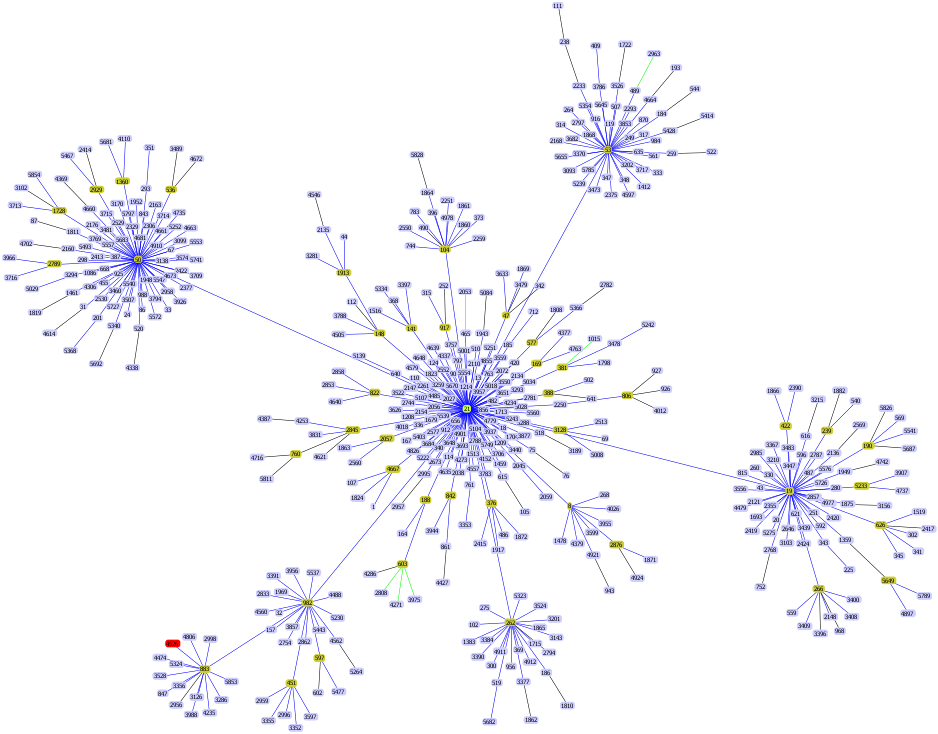

Supplement: Figure S2 — The goeBURST analysis of C. jejuni CC-21. A total of 547 STs belonging to the CC-21 were used to analyze possible patterns of their evolutionary descent. (TIF) [file pone.0048394.s002.tif]

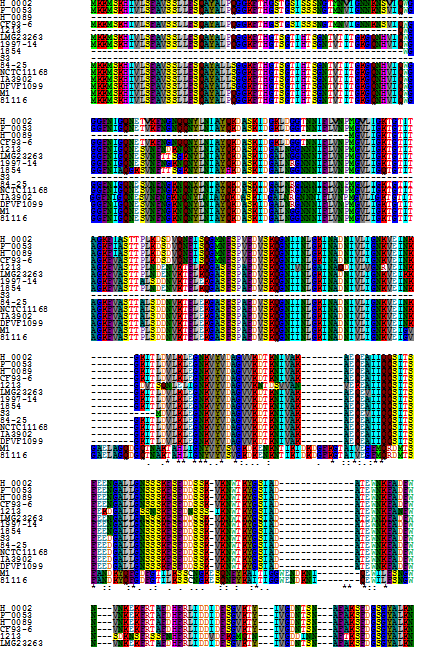

Supplement: Figure S3 — Multiple alignment of putative filamentous hemagglutination domain protein (FHA) in C. jejuni ST-4526 representative isolates (H_0002, H_0089, P_0053), and those from 12 other C. jejuni strains (CF93-6, 1213, LMG23263, 1997-14, 1854, S3, 84-25, NCTC11168, IA3902, DFVF1099, M1, and 81116). The conserved domain Architecture Retrieval Tool (CDART) (http://www.ncbi.nlm.nih.gov/Structure/exington/lexington.cgi) predicts the putative hemagglutination activity domain at 22 to 135 aa from the N-terminus of the H_0002 isolate (shown in bold). (TIF) [file pone.0048394.s003.tif]

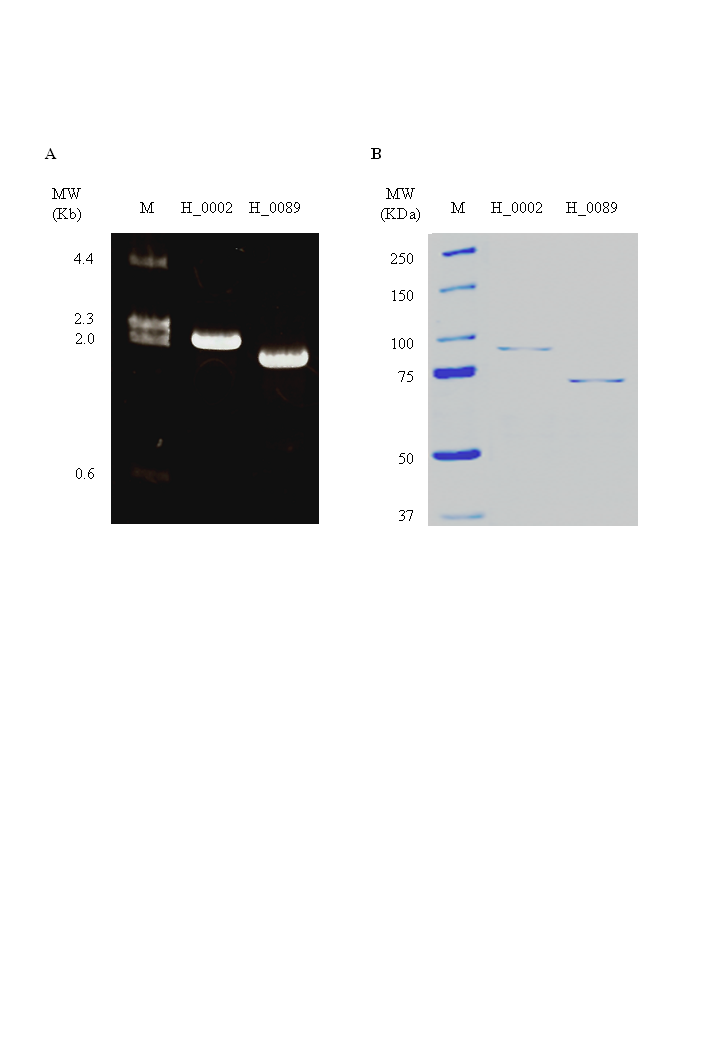

Supplement: Figure S4 — Cloning and expression of the recombinant filamentous hemagglutination domain protein (rFHA) from C. jejuni H_0002 and H_0089. The putative FHA-encoding gene was PCR-amplified (section A), and ligated into pBAD202 D-Topo vector. Expression was induced under the control of L-arabinose in E. coli LMG194 strain. Both the purified rFHA proteins were loaded onto 10% acrylamide gel, visualized by CBB stain. M, molecular markers (section A, λ/HindIII; section B, Bio-Rad Precision plus Protein standard Dual color). (TIF) [file pone.0048394.s004.tif]

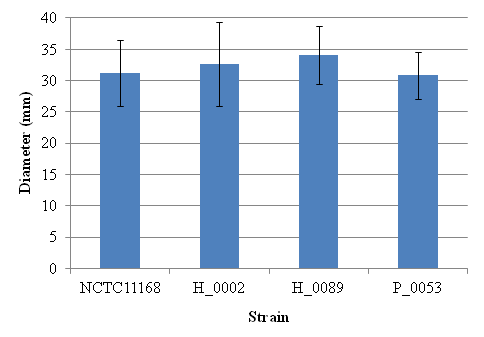

Supplement: Figure S5 — Motility of C. jejuni ST-4526 isolates on soft agar plates. 5 µl aliquots of C. jejuni ST-4526 isolates (H_0002, H_0089, P_0053) and NCTC11168 cultures were spotted on 0.4% soft agar plates. At 24 h post incubation, the diameters of halo around the spot were measured. The data showed means ± standard deviation (SD) from three independent testing. (TIF) [file pone.0048394.s005.tif]
